# Frontotemporal dementia and amyotrophic lateral sclerosis: revisiting one of the first case reports with neuropathology examination

**DOI:** 10.1590/S1980-57642014DN81000013

**Published:** 2014

**Authors:** Ricardo Nitrini

**Affiliations:** Behavioral and Cognitive Neurology Unit, Department of Neurology, and Cognitive Disorders Reference Center (CEREDIC). Hospital das Clínicas of the University of São Paulo School of Medicine, São Paulo, Brazil.>

**Keywords:** dementia, frontotemporal dementia, amyotrophic lateral sclerosis, neuropathology, motor neuron disease

## Abstract

The occurrence of dementia in amyotrophic lateral sclerosis (ALS) was only widely recognized in the late 20^th^ century. Hitherto, it was believed that dementia was a rare event due to the fortuitous association with other diseases. In 1924, Kostantin Nikolaevich Tretiakoff and Moacyr de Freitas Amorim reported a case of dementia with features of frontotemporal dementia (FTD) that preceded the motor signs of ALS. Neuropathological examination confirmed ALS and found no signs of other dementia-causing diseases. The authors hypothesized that dementia was part of ALS and recommended the search for signs of involvement of motor neurons in cases of dementia with an ill-defined clinical picture, a practice currently accepted in the investigation of cases of FTD. This was one of the first descriptions of dementia preceding the motor impairments of ALS and was published in Portuguese and French in Memórias do Hospício de Juquery.

## Abstract (pt)

A ocorrência de demência na esclerose lateral amiotrófica (ELA) somente foi amplamente reconhecida no final do século 20. Antes disso, acreditava-se que demência era ocorrência rara que se devia à associação fortuita de outra doença. Em 1924, Kostantin Nikolaevich Tretiakoff e Moacyr de Freitas Amorim relataram caso de demência com as características de demência frontotemporal (DFT) que precedeu os sinais motores da ELA. O exame neuropatológico confirmou ELA e não encontrou sinais de outras doenças que cursam com demência. Os autores do artigo aventaram que a demência fazia parte da ELA e recomendaram a investigação da presença de sinais de comprometimento dos neurônios motores em dementes com alterações cognitivas e comportamentais mal definidas, conduta atualmente aceita na investigação de casos de DFT. Esta foi uma das primeiras descrições de demência precedendo a síndrome motora da ELA e foi publicada em português e em francês em Memórias do Hospício de Juquery.

## INTRODUCTION

In the original description of amyotrophic lateral sclerosis (ALS),^1^ Charcot made no reference to cognitive or behavioral disturbances.^2,3^

Although Pierre Marie had described "undeniable depression of mental faculties in all cases" (p. 486),^4^ the presence of cognitive or behavioral impairment in ALS was only widely recognized as relatively common in the last quarter of the twentieth century.^5,6^ Hitherto, ALS or motor neuron disease (MND) associated with dementia was suggestive of a rare variety of disease that affected the Chamorros who inhabit the island of Guam,^7^ or a very rare (and even questionable) type of Creutzfeldt-Jakob disease called amyotrophic form.^8^

From the 1970s, some authors, especially in Japan,^2,9^ but also in Western countries,^10,11^ began describing dementia accompanied by signs of MND that sometimes had the characteristics of ALS and at other times of spinal muscular atrophy.

Gradually, it became clear that the association of MND with dementia, mainly frontotemporal dementia (FTD), is a relatively common entity. However, in the 1990 international consensus conference for establishing diagnostic guidelines of ALS held in El Escorial, Spain,^12^ the presence of cognitive alterations was still considered an exclusion criterion.^13^ A few years later, in the first attempt to classify the FTDs promoted by groups of Swedish and British researchers, from Lund and Manchester, respectively, FTD associated with MND was finally included as one of the forms of FTD.^14^

The purpose of this article is to draw attention to a case report of ALS with dementia published in Brazil in 1924, in both Portuguese^15^ and French,^16^ with clinical and pathological evaluations.

## SUMMARY OF THE CASE

In June 1922, a 25-year-old woman, with elementary education, was admitted to the Juquery Hospice, located in the city of Franco da Rocha, in Greater São Paulo, with a diagnosis of "*dementia praecox*". Her mental state was characterized by "absolute indifference to everything around her and by herself"; "complete absence of affective feelings", "incapable of any initiative", "unmotivated laughter and cries." "Memory was null and association of ideas was done extravagantly." "She walked through the courtyard next to the other patients, without any other activity". (pages 81^15^ and 259^16^).

She was in poor health, with unremitting dysentery, skin pallor and tachycardia. Her general condition was declining, with loss of weight. Approximately one year after admission she became bedridden, when generalized muscle atrophy became severe, predominantly in the lower extremities, which were also more weakened in relation to the upper limbs. Babinski sign was present on her left side, and tendon reflexes were diminished in the lower and upper limbs. Fasciculations were absent. She developed dysphagia and death occurred in October 1923.

Pathological examination revealed ulcerative intestinal tuberculosis. There was also slight subacute myocarditis and mild nephritis. At neuropathological examination, no changes were evident in the meninges and cerebral vessels. Systematic degeneration of the pyramidal tract (visible up to the level of the mesencephalic peduncles), chromatolysis of pyramidal cells of Betz, marked chromatolysis of numerous cells of the anterior horn of the cervical and lumbar cord, were found (Figure). The lesions of the anterior horn cells predominated in the outer side of the spinal cord and spared the anterior horn cells of the thoracic spinal cord. The authors also reported that they found no atrophy of the frontal cortex.

**Figure:**
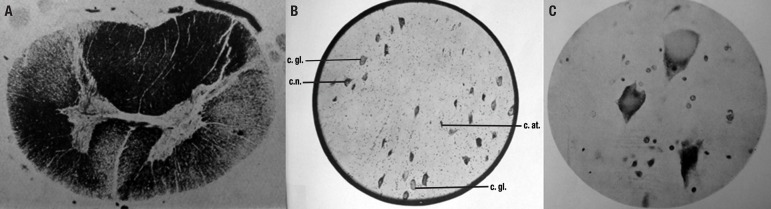
[A] Spinal cord, thoracic level. Degeneration of the direct and crossed pyramidal tracts. Weigert; [B] Anterior column of the spinal cord. Numerous neurons presenting chromatolysis. Cresyl-violet. c.n.: normal cell; c.at.: atrophied cell; c.gl: globoid cell; [C] Ascending frontal gyrus. Chromatolysis of Betz cells. Cresyl-violet.

## TRETIAKOFF AND AMORIM'S HYPOTHESIS

The authors classified the neurological disease as ALS, more specifically as a pseudopolyneuritic form of ALS or Patrikios' form, which starts in the lower limbs causing a reduction or abolition of deep tendon reflexes.^17^ They did not rule out that the action of toxins or anemia related to the tuberculosis may have contributed to the muscle atrophy, but the association with intestinal tuberculosis seemed fortuitous to them.

They drew attention to the early occurrence and intensity of "mental disorders" in their case, but reminded that Pierre Marie had insisted long ago that psychiatric disorders were frequent in ALS. However, the changes described by Pierre Marie occur in more advanced stages of ALS, contrary to what was observed in their case.

Although the neuropathological examination did not reveal cortical lesions besides those of the Betz cells, Tretiakoff and Amorim stated that "the intensity of the mental phenomena leads us to believe in its existence". (pages 89^15^ and 264^16^)

They concluded by proposing that "it would be helpful to research similar spinal injuries in some demented that show ill-defined paretic disturbances that are ordinarily attributed to the cachectic condition of the patients or even to their mental disorders" (pages 90^15^ and 265^16^). By "paretic disturbances" the authors were referring to the behavioral and cognitive changes occurring in paretic neurosyphilis.

## WHAT WAS SO SPECIAL ABOUT THIS CASE REPORT?

This publication has at least three special features.

Firstly, the description of the cognitive and behavioral symptoms is suggestive of frontotemporal dementia (FTD), characterized by apathy, loss of initiative and lack of affection. But there were also unmotivated laughter and groans, an odd association of ideas and memory described as null. These manifestations are suggestive of FTD with the exception of the involvement of memory, which is not characteristic of this disorder in its early stages, but occurs at a more advanced stage. Given the patient was hospitalized with severe dementia, the diagnosis of FTD is likely in this case.^18^ It is noteworthy that the motor signs of ALS appeared later in the evolution of the disease in this case. No information about the occurrence of a similar disease in relatives of the patient was available, but a genetic form of ALS is possible, especially in view of the early-onset of the disease.^19^ It is likely that this was one of the first descriptions with clinical and neuropathological evaluations of FTD associated with ALS.

Clinical descriptions of psychic, behavioral and cognitive disorders in ALS had previously been reported by several authors.^3,20,21^ In many of these cases, post-mortem histopathological examination was not performed and the possibility of other diseases, such as neurosyphilis^3,21^ or senile dementia,^2^ were entertained. Case reports of ALS with mental symptoms submitted to neuropathological examination were scarce before the publication of this case in 1924. In two reviews, Mitsuyama et al.^9^ and Zago et al.^14^ cited no papers with neuropathological examination published prior to 1932. In the review of 34 Japanese cases by Morita et al.,^3^ the earliest case had been published in 1935.22 However, Gerber and Naville (1921) reported a case with cortical degenerative changes in a man with ALS manifesting late mental symptoms.^23^

After the publication of this case, van Bogaert (1925)^21^ published a case series of 12 cases of ALS with mental symptoms. However, only one case had histopathological evaluation, which revealed a lacunar state in the basal ganglia. In 1932, Wechsler and Davison reported three cases with mental symptoms and degenerative changes mainly in the frontal and temporal cortex, and concluded that in their cases and a few others, mental symptoms were due to cortical degenerative changes associated with ALS.^2^

Secondly, Tretiakoff and Amorim's conclusion that it would be useful to search for similar spinal cord injuries (or symptoms of involvement of lower motor neurons) in patients with FTD is currently a well-established procedure in clinical and neuropathological evaluations. The absence of other cortical pathological changes typical of FTD may be explained by the fact that the neuropathology of FTD with or without MND had been elusive for many years and, until recently, most FTD cases were classified as dementia lacking distinctive histologic features.^24^ With immunohistochemistry for specific proteins, the neuropathological diagnosis of FTD is now far more straightforward.^25^

And finally, this was a clinical and neuropathological study conducted in Brazil and published simultaneously in French and Portuguese in a Brazilian journal - Memórias do Hospício de Juquery which, after its second year, changed its name changed to Memórias do Hospital de Juquery. In the first issue of the journal, its founder told the story of the creation of the pathology laboratory and how bringing an experienced neuropathologist like Tretiakoff^26^ to Brazil was made possible who, in his doctoral thesis in 1919, had concluded that the substantia nigra was consistently affected in parkinsonism.^27^ For several years, the articles continued to be published bilingually in Portuguese and also in French or German, languages that had great prominence in neurosciences at the time, but the journal ceased to exist in 1935. The quality of published articles, in particular of the neuropathological studies, warrants bringing the contributions of Tretiakoff and Memorias do Hospital de Juquery to the attention of Brazilian neuroscience.^28^
